# Supplementary figures and images for: Establishment of the body condition score for adult female Xenopus laevis
Source: PLoS One. 2023 Apr 26;18(4):e0280000. doi: 10.1371/journal.pone.0280000 (PMC10132665; doi:10.1371/journal.pone.0280000)

Chart of Body Condition Score for adult female *Xenopus laevis*


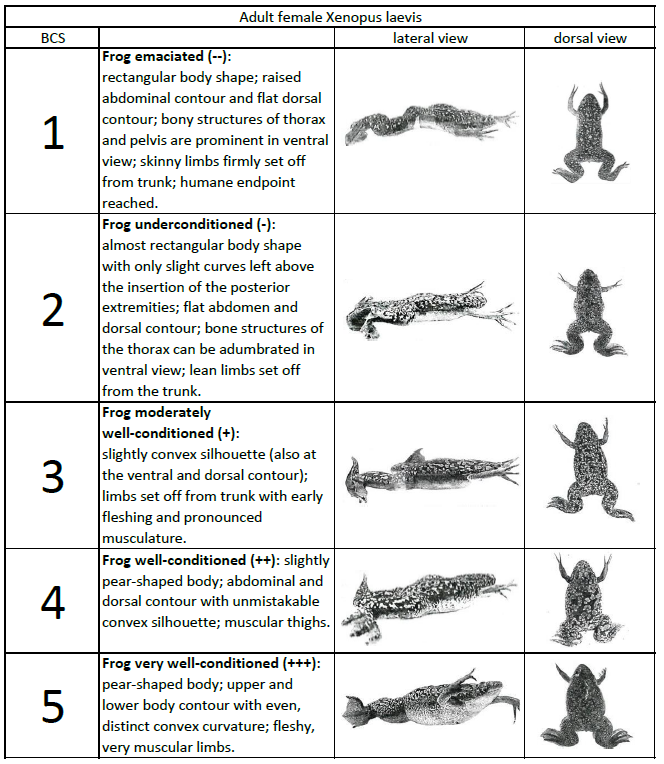

Supplement: S1 Fig — Chart of Body Condition Score for adult female Xenopus laevis. (DOCX) [file pone.0280000.s001.docx]
