# Supplementary material for: Establishment of the body condition score for adult female Xenopus laevis
Source: PLoS One. 2023 Apr 26;18(4):e0280000. doi: 10.1371/journal.pone.0280000 (PMC10132665; doi:10.1371/journal.pone.0280000)
Supplement: S1 File — Housing conditions were analyzed with linear regression, in which the dependent variable (BCS) was modeled as a function of the housing condition. (DOCX) [file pone.0280000.s002.docx]

In the present study, animals of the following three different housing conditions were evaluated: large circulating, semi-closed circulation system (17 tanks) with water preparation and conditioning to 21°C ± 1°C (circulation large); small circulating, semi-closed circulation system (4 tanks) with water preparation and conditioning to 20°C ± 1°C (circulation small), individual fresh water tanks (240l tank) without water preparation at 12°C ±3°C (cold water flow-through).

The housing conditions were analyzed with linear regression, in which the dependent variable (BCS) was modeled as a function of the housing condition. The “cold water flow-through” factor was defined as the intercept level in the regression analysis.


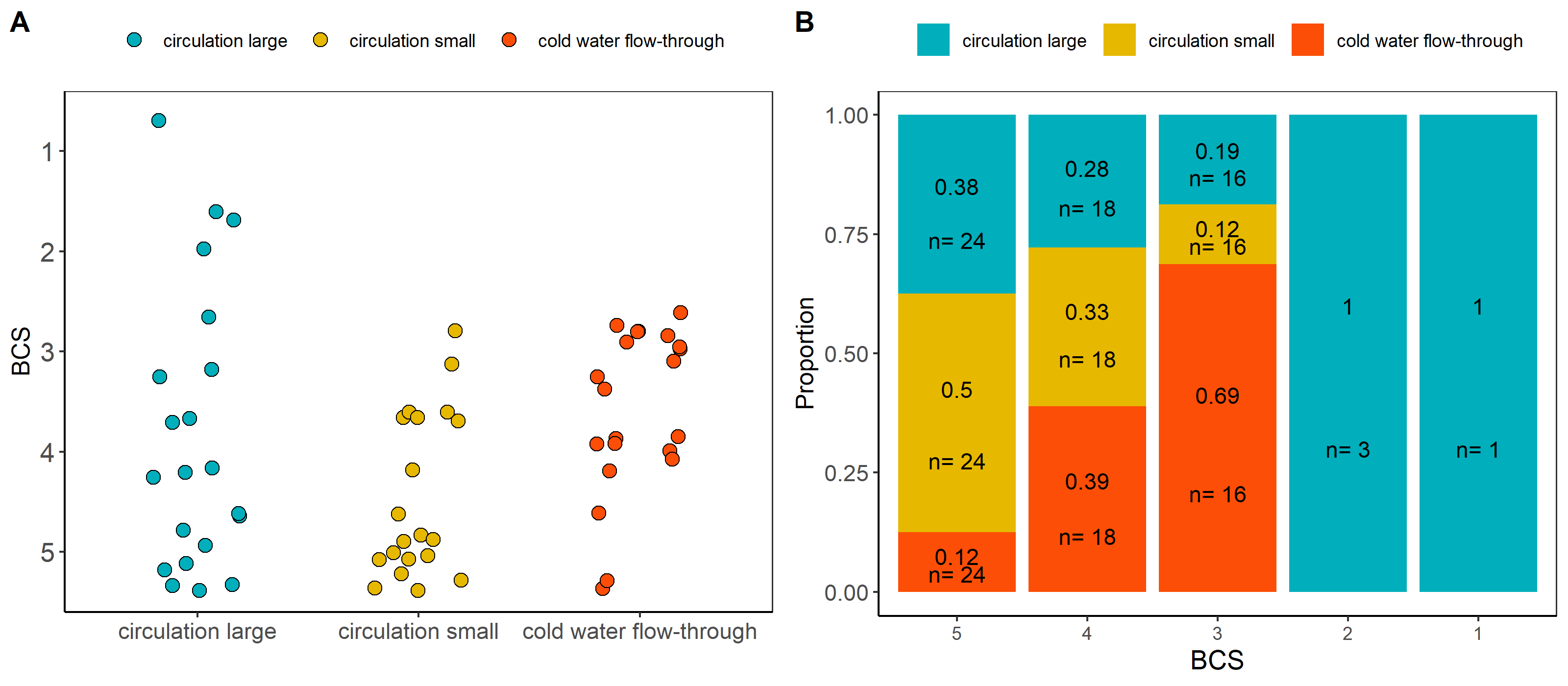


Table 4. Result of the linear regression.

The linear regression shows that the “tempered circulation system small” significantly impacts the BCS. In comparison to the “cold water flow-through”, the BCS is 0.88 points lower on average (β_small_=-0.88, SE=0.3, p=0.004), while the “circulation large” shows no difference to the cold water system.

Since the linear regression showed significant coefficient effects, the corresponding ANOVA was also significant (F(2,59)=4.75, p=0.012). Subsequent post hoc tests revealed more specific between-housing condition differences.

The post hoc test shows that the “circulation small” vs. “circulation large” group was not significant (p_adj_=0.08). However, the difference in average BCS was substantial (∆=-0.64), and there was some evidence of a potential effect (p<0.1). While the comparison of “cold water” vs. “circulation large” was not significant (p=0.69) and showed a much lower difference in the average BCS (∆=0.24), the “cold water flow-through” system showed a significant BCS difference (∆=0.88, p=0.01). The post hoc tests were multiplicity-adjusted with the Tukey method.
