# Supplementary material for: Establishment of the body condition score for adult female Xenopus laevis
Source: PLoS One. 2023 Apr 26;18(4):e0280000. doi: 10.1371/journal.pone.0280000 (PMC10132665; doi:10.1371/journal.pone.0280000)
Supplement: Attachment — Submitted filename: Response to Reviewers.docx [file pone.0280000.s003.docx]

**Response letter PONE-D-22-22653**

Dear Michael Klymkowsky,

First of all, we would like to thank the Editors and the Reviewers for their valuable input and their pro bono work.

We tried to meet the suggestions of the reviewers and hope that this improved the quality of our manuscript, accordingly. In addition, our response to the individual comments of the reviewers are provided in this point-to-point response letter.

**Referee(s)' Comments to Author:**

**Reviewer #1:**

1. **Since the authors used animals from three different housing systems, it might be useful to include this information in the counts of weights and BCS and whether there was any effect of housing on BCS (maybe include housing origin in Table 1?). The small numbers of BCS1-2 animals might make any conclusions difficult, but there might be useful information on any differences in weight or BCS3-5 across the housing conditions.**

*We thank you for this valuable feedback and appreciate the comment. For this purpose, we have compared the three different housing conditions and deposited the corresponding data in the supplemental of the manuscript.*

*At this point, we would like to point out again that the husbandry conditions per se, were not the focus of the paper and the influence on the physiology of the animals cannot be addressed here in detail.*

*However, to make the data and graphs transparent and comprehensive to the reader, we have provided further explanations in the supplement.*

*Please see the reference to the supplemental part of the manuscript in lines 240 - 241:*

*“The distribution of assessed BCS within the different housing conditions and their effects on BCS are provided in the supplemental materials (supplemental 2).”*

*Please see here what we added to the supplements concerning your feedback:*

*“In the present study, animals of the following three different housing conditions were evaluated: large circulating, semi-closed circulation system (17 tanks) with water preparation and conditioning to 21°C ± 1°C (circulation large); small circulating, semi-closed circulation system (4 tanks) with water preparation and conditioning to 20°C ± 1°C (circulation small), individual fresh water tanks (240l tank) without water preparation at 12°C ±3°C (cold water flow-through).*

*The housing conditions were analyzed with linear regression, in which the dependent variable (BCS) was modeled as a function of the housing condition. The “cold water flow-through” factor was defined as the intercept level in the regression analysis.*

*Table 4. Result of the linear regression.*

*The linear regression shows that the “tempered circulation system small” significantly impacts the BCS. In comparison to the “cold water flow-through”, the BCS is 0.88 points lower on average (β_small_=-0.88, SE=0.3, p=0.004), while the “circulation large” shows no difference to the cold water system.*

*Since the linear regression showed significant coefficient effects, the corresponding ANOVA was also significant (F(2,59)=4.75, p=0.012). Subsequent post hoc tests revealed more specific between-housing condition differences.*

*The post hoc test shows that the “circulation small” vs. “circulation large” group was not significant (p_adj_=0.08). However, the difference in average BCS was substantial (∆=-0.64), and there was some evidence of a potential effect (p<0.1). While the comparison of “cold water” vs. “circulation large” was not significant (p=0.69) and showed a much lower difference in the average BCS (∆=0.24), the “cold water flow-through” system showed a significant BCS difference (∆=0.88, p=0.01). The post hoc tests were multiplicity-adjusted with the Tukey method.*

2. **Other organisms often have a unified chart showing the BCS scores next to the views describing the condition criteria. It would be good to include such a chart for people to print out and refer to (maybe as a supplemental figure). Maybe also include in the score descriptions terminology found in other charts "underconditioned", "well-conditioned", etc.**

*We are very grateful for the positive review of our manuscript and addressed your remarks accordingly. Therefore, we attached an overview chart of the BCS to the paper's new version as a supplementary figure, enabling working groups to make further use of it. We have also addressed your suggestion and modified the description about the terminology used in previously published BCSs.*

*Please see the revised manuscript lines:*

*Line 177-178: “…and a BCS 5 is regarded as a very well-conditioned physical state.”,*

*Line 180-181: „…The following assessment criteria are distinguished and defined for the respective BCS levels (Supplemental figure 1).“,*

*Line 187: “Frog underconditioned (-): …” and*

*Line 197: “Frog very well-conditioned (+++): …”*

*Supplemental figure 1: Chart of Body Condition Score for adult female Xenopus laevis*

**3. Minor typos/word usage suggestions:**
*Thank you for pointing out typos in the manuscript, which we have corrected, accordingly. Please see the revised version of the manuscript in the appropriate places.

line 42; common name used for X. tropicalis changed to "western clawed frog".
line 50; repeat use of term 'undemanding' changed to 'unpretentious'
line 56; ... live 'purely aquatically'
line 305; 'was intended' to be used ....
line 343/344; 'coelomic cavity'*

**Reviewer #2:**

1. **The age of the frogs is not presented. The authors do not state if the frogs are all the same age and from the same clutch. How different are the ages and are they all siblings? This information would be helpful.**

*We apologize that this information was not sufficiently addressed in the paper. We have tried to obtain appropriate data and breeding information from the breeder. Unfortunately, they do neither record the individual age, date of spawning nor any relationship of the animals. Therefore, unfortunately, no further information can be given. We have indicated this in line 146/147 of the manuscript.*

*“However, no statement can be made about the age or the degree of relationship of the animals.”*

*However, we will gladly take up this point for investigations within the scope of subsequent projects on this topic in order not to neglect these possible influencing factors.*

2. **Since Xenopus females are used for oocyte and eggs how does the BCS correlate to the quality of oocytes and eggs? Does BCS impact the quality? Any effect on in vitro fertilization. This information would make BCS score more appropriate for the research community. As it stands the current BCS information would only pertain to those keeping the frogs as pets.**

*Thank you for this interesting and important comment. We are also investigating the significance of the BCS for research. The interpretation of the BCS concerning egg and oocyte quality will be the focus of further investigations and independent tests. We will, therefore, pursue this approach with great interest and publish additional data in due time.*

*Currently, the BCS is seen as a purely descriptive tool for daily animal care and routine examination of individual animals within laboratory animal husbandry.*

*As we are aware of this limitation of the BCS, we addressed this topic in lines 395-398 in the revised manuscript:*

*“However, what effect the age of the animals has on the occurrence of the respective BCS groups and what influence the BCS might have on the quality of oocytes and eggs cannot be addressed at this time. It also remains to be investigated to what extent the BCS for female Xenopus laevis can be transferred to male animals. “*

**What about male frogs?**

*We are also willing to consider this criticism and examine the transferability of the BCS we presented for female animals to males. However, since these animals are much less frequently kept and used for research on eggs and oocytes, this cannot be answered at this stage.*
