# Supplementary material for: Establishment of the body condition score for adult female Xenopus laevis
Source: PLoS One. 2023 Apr 26;18(4):e0280000. doi: 10.1371/journal.pone.0280000 (PMC10132665; doi:10.1371/journal.pone.0280000)
Supplement: Attachment — Submitted filename: Response to Reviewers_R1.docx [file pone.0280000.s004.docx]

**Response letter PONE-D-22-22653R1**

Dear Sir,

Dear Mr. Klymkowsky,

First of all, we would like to thank the Editors and the Reviewers again for their valuable input and their pro bono work.

We are sorry that our response to Reviewer 2's comments was not sufficient for him and are of course fine with the request to include the suggested passage in the manuscript.

**Referee(s)' Comments to Author:**

**Reviewer #2:**

1. **The authors should include the following statement in the manuscript that they wrote in response to my review:**

**“the BCS is seen as a purely descriptive tool for daily animal care and routine examination of individual animals within laboratory animal husbandry.”**

We regret that our response to your comment was not sufficient in view of the reviewer. We followed your advice and therefore included the statement you suggested in the manuscript.

Please, find the text section to be inserted in lines 392 – 394:

*“Therefore, the BCS is seen as a purely descriptive tool for daily animal care and routine examination of individual animals within laboratory animal husbandry.”*

2. **Also the abstract text should be slightly modified. In line 24-25 sentence in abstract the authors state the aim was to establish the BCS in terms of experimental and housing refinement. The word experimental should be removed since there is no correlation made between BCS score and experimental use of Xenopus.**

In addition, we have adjusted the abstract as requested and removed the term “experimental”. However, in order to preserve the laboratory animal science background at this point, we have modified the information accordingly. We hope that this more correct wording is in line with your intentions.

The corresponding section of the abstract now reads as follows (lines 24 - 25):

*“The present study aimed to establish a species-specific BCS for clawed frogs in terms of housing refinement in lab-animal facilities.”*
